# Editorial: Ionic liquids and deep eutectic solvents: Two contrasting options or opposite sides of the same coin?

**DOI:** 10.3389/fchem.2023.1169688

**Published:** 2023-02-27

**Authors:** Lorenzo Guazzelli, Isabel Maria Marrucho, Tiancheng Mu, Tamal Banerjee

**Affiliations:** ^1^ University of Pisa, Pisa, Italy; ^2^ Higher Technical Institute, University of Lisbon, Lisbon, Portugal; ^3^ Renmin University of China, Beijing, China; ^4^ IIT Guwahati, Guwahati, India

**Keywords:** ionic liquids, deep eutectic solvents, green chemistry, carbon dioxide, batteries, electrochemical deposition

Ionic liquids (ILs) and deep eutectic solvents (DESs) are reported as the alternatives to traditional volatile organic compounds (VOCs). Both ILs and DESs are thought to be tunable and designable although there are still some disagreements and disputes on the nature, greenness, stability of ILs and DESs and even on the relationship between them (Chen and Mu, 2021; Zhang et al., 2022; Afonso et al., 2023). In this Research Topic “*Ionic liquids and deep eutectic solvents: Two contrasting options or opposite sides of the same coin?*”, we have collected four articles (including two original researches and two reviews) in total, representing the recent intermolecular, interactions, physical properties and relevant applications for ILs and DESs. Below, we would like to provide the summary and research highlight for theses four articles.

Low melting temperature and high ionic conductivity are the key physical properties for ILs. Low melting temperature could make ILs show liquid state at low temperature, while high ionic conductivity is very helpful for ILs to show favorable performance for electrochemical catalysis and batteries. Unfortunately, ionic conductivity of ILs tends to be low when the temperature is low. One of the strategies to solve this contradiction is to design ILs with much lower melting temperature to keep ILs liquid for the purpose of achieving high ionic conductivity. Abdurrokhman and Martinelli (Abdurrokhman I and Martinelli) found that high ionic conductivity and low melting point (and thus wide temperature range of liquid state) could be achieved simultaneously by the strategy of mixing two kinds of ILs with the same imidazolium cation and two different kinds of anions. Binary ILs could also keep the low volatility and high stability of ILs in addition to high ionic conductivity and low melting point, which would be more favorable than the mixtures of VOCs + ILs reported in many reports. This strategy would be very helpful for the application of ILs in electrochemical catalysis and batteries.

Traditional electrochemical deposition of aluminium is commonly performed at very high temperature thus it is energy-intensive and releases carbon dioxide (CO_2_) and corrosive hydrogen fluoride. Wu et al. make a summary on the electrochemical deposition of aluminium by ILs-based electrolytes. Furthermore, ILs are regarded as tunable, green and safe solvents for the electrochemical deposition of aluminium at mild temperature. These specific fields of aluminium deposition include nano-aluminium, high-purity aluminium and rechargeable aluminium ion batteries. Finally, the authors suggested that in-depth investigation should be conducted to establish the technical criterial.

CO_2_ capture and separation is very important for CO_2_ mitigation, fuel purification and CO_2_ conversion. Available technologies (e.g., absorption, adsorption, membrane and cryogenic separation) for CO_2_ capture and separation are expensive, energy-consuming, corrosive or non-sustainable. Foorginezhad et al. give a review on the CO_2_ capture by ILs and DESs due to their unique properties. ILs and DESs could interact with CO_2_ *via* physical or chemical interaction, while the unavailable properties of ILs and DESs could be estimated by COSMO-RS for the further improvement of CO_2_ capture. Criteria are established for evaluating the CO_2_ absorption *via* physical interaction (i.e., capacity, viscosity and selectivity) and chemical interaction (capacity, viscosity and absorption enthalpy). According to these criteria, top 10 ILs and DESs are suggested as the most promising green solvents for CO_2_ absorption.

Immobilization technology by developing immobilized-ILs sorbents could improve the capacity and selectivity for CO_2_ capture and separation while avoiding the usage of VOCs; however, huge amount of ILs (∼10^18^) synthesized by trial and error would be time-consuming and low-efficient. Therefore, it is necessary to develop theoretical methods to predict the efficiency of CO_2_ capture by ILs before the experiment. Dai et al. for the first time propose that compressibility of ILs could be used as the new index for electrolyte perturbed-chain statistical associating fluid theory to predict CO_2_ capture by immobilized-ILs. Furthermore, desorption enthalpy of CO_2_ from ILs could also be identified. This theoretic model is reliable, efficient and consistent with the experiments results.

We hope this Research Topic could help better understanding the intermolecular interaction, physical property and application of ILs and DESs on the goal of replacing VOCs. It should be noted that not all ILs and DESs are green and cheap, which mainly depends on the selection of appropriate raw materials and interaction *via* reasonable design. Finally, we thank all the authors, reviewers, and editors for their contribution in the Research Topic of “*Ionic liquids and deep eutectic solvents: Two contrasting options or opposite sides of the same coin*?”.
